# Linc01513 inhibits the malignant potential of Nasopharyngeal carcinoma by binding to PTBP1

**DOI:** 10.7150/jca.62112

**Published:** 2021-10-28

**Authors:** Juan Wang, Xiaolan Cai, Liqiang Zhang, Dapeng Lei

**Affiliations:** 1 Department of Otorhinolaryngology, Qilu Hospital of Shandong University, Jinan, China; 2 NHC Key Laboratory of Otorhinolaryngology, Shandong University, Jinan, China

**Keywords:** linc01513, Nasopharyngeal carcinoma, proliferation, invasion, PTBP1

## Abstract

LncRNAs are reported to be involved in tumor proliferation, invasion and metastasis, and are considered as potential biomarkers and therapeutic targets for human cancer, including head and neck cancer. In this study, we screened the differentially low-expressed linc01513 by bioinformatic to detect its expression and biological effect on nasopharyngeal carcinoma (NPC). MTT was used to evaluate the effect of linc01513 on the proliferation of NPC cells. Wound healing assay was used to determine the cells migration ability. The matrix transwell was used to further detect the role of linc01513 in cell invasion. Western blot was used to detect the expression of epithelial-mesenchymal transformation (EMT)-induced transcription factors E-cadherin, vimentin and Slug. The results showed that silence of linc01513 could promoted the proliferation, migration and invasion of NPC cells. The *in vivo* experiment showed that overexpression of linc01513 could inhibit the volume and weight of xenograft tumors. Database prediction, RNA pull-down and RIP experiments suggested that linc01513 may play an anti-tumor effect by inhibiting PTBP1 protein level. It is suggested that linc01513 directly binds to PTBP1 protein and mediates the EMT process and malignant biological behavior of NPC cells, which provides a new molecular marker for the prognosis and treatment of NPC.

## 1. Introduction

Nasopharyngeal carcinoma (NPC) is a malignant head and neck tumor that occurs most commonly in Southeast Asia and North Africa, with distinct regional characteristics and different pathogenesis 1, 2. Because of the unique biological and anatomical characteristics of nasopharyngeal carcinoma, radiotherapy has been the main and primary treatment. Although IMRT and concurrent chemoradiotherapy significantly improve the control rate of nasopharyngeal carcinoma, local recurrence and distant metastasis are still the main reasons for current treatment failure of nasopharyngeal carcinoma 3-5. Therefore, it is necessary to explore the molecular mechanism of invasion and metastasis of nasopharyngeal carcinoma and find effective therapeutic targets.

Long non-coding RNA (lncRNA) is a kind of non-coding RNA with a length of more than 200 nucleotides, and its protein-coding ability is extremely limited 6. More and more studies have shown that lncRNA can regulate tumor progression through various mechanisms such as epigenetic regulation, gene transcription and post-transcription regulation 7, 8. Although studies have shown that lncRNA plays a significant role in the process of nasopharyngeal carcinoma, such as HOTAIR, MALAT1 and ANRIL, the expression characteristics and functional mechanism of most lncRNA in nasopharyngeal carcinoma still need to be further explored^9^, ^10^. The mechanisms of lncRNAs are diverse, including transcriptional regulation and post-transcriptional gene expression regulation, epigenetic transcriptional regulation, such as competitive endogenous RNAs, chromatin remodeling and histone modification as miRNA sponges^11^. Epigenetic regulation of target genes is the main function of the research of lncRNAs. Studies have shown that a novel lncRNA LINC01133, which is down-regulated by TGF-β, inhibited epithelial-mesenchymal transition (EMT) and metastasis in colorectal cancer (CRC) cells. Splicing factor SRSF6 directly interacted with LINC01133, and SRSF6 promotes EMT and metastasis of CRC cells independent of LINC01133. That study confirmed that the EMT process of CRC cells is regulated by LINC01133 and dependent on the presence of SRSF6^12^.

Invasion and metastasis of tumor cells to surrounding tissues and distant organs are important characteristics of malignant tumors 13. The metastatic routes of nasopharyngeal carcinoma include cervical lymph node metastasis, local invasion metastasis and hematogenous metastasis. In the last 30 years, the transformation of epithelial cells into mesenchymal phenotype, also known as mesenchymal transformation, has played a key role in embryonic development and tumor progression, as well as many physiological and pathological processes. Studies have reported that LncRNAs promote the migration and invasion of tumor cells by inducing EMT^14^. There are studies that have reported lncRNA CCAT2 by inducing epithelial ectomesenchymal transformation to promote bile duct cancer cell migration and invasion^15^; LncRNA-ENST00000434223 through Wnt/β-catenin signaling pathway to inhibit gastric cancer cell proliferation, invasion and EMT^16^. However, the exact mechanism is still not thoroughly studied. Therefore, the study of the role of research LncRNAs in nasopharyngeal carcinoma and its mechanism is an urgent problem to be solved.

Linc01513 differentially expressed in nasopharyngeal carcinoma was screened from GEO database. The purpose of this study was to investigate the expression of linc01513 in nasopharyngeal carcinoma tissues and nasopharyngeal carcinoma cells and its biological effect on nasopharyngeal carcinoma cells.

## 2. Material and method

### 2.1 Tissue specimen

The surgical specimens of 58 patients with definite pathological diagnosis and adjacent nasopharyngeal epithelial tissue samples were collected from Department of Otorhinolaryngology, Qilu Hospital of Shandong University, patients with other connective tissue diseases and malignancies were excluded. Clinicopathologic features of patients were shown in Table 1. The experiments were permited by the Ethics Review Committee of Qilu Hospital of Shandong University and the patients signed informed consent.

### 2.2 Animals

Animal experiments were permited by the Animal Protection and Ethics Committee of Qilu Hospital of Shandong University. BALB/c nude mice (6-8 weeks) were purchased from Beijing Weitong Lihua Experimental Animal Technology Co., Ltd. (Beijing, China). For the experiment of Xenograft, 6-10B cells (5 × 10^6^) were suspended in 200 μL saline and injected subcutaneously.

### 2.3 Cell culture

6-10B and SUNE-1 cell lines and stable-transfected cells were purchased from CHI Scientific, Inc (Jiangsu, China). The cells were cultured with complete medium including 89% 1640 and 10% FBS, both were purchased from Biological Industries (Beit-Haemek, Israel), and maintained in incubator with 37°C and 5% of CO_2_ saturated humidity.

### 2.4 Transfection

The 6-10B and SUNE-1 cells were plated until the cell density reached 80% confluency of dishes to transfect. Smart silencer of linc01513 and siRNA of PTBP1 were constructed by Ruibo (Guangzhou, China) and transfected with Lipofectamine 2000 (Invitrogen, Carlsbad, CA).

### 2.5 Western blot

After RIPA cleavage, we extracted total protein and measured with BCA method. After quantitative denaturation, protein electrophoresis membrane transfer and blocked with 5% Skim milk. The first incubation and second incubation were carried out. The expression of the protein was expressed by the gray value. The first antibody of PTBP1 (1:300), E-cadherin (1:500), vimentin (1:500) and Slug (1:300) were purchased from ProteinTech (Wuhan, China).

### 2.6 Biotin-RNA pull-down

First, RNA was pretreated to form a secondary structure, and then total cellular proteins were extracted. The total protein was first preincubated with 60 μL streptavidin coated with lytic solution, and the lipopolysaccharide column (Streptavidin beads), was rotated slowly at room temperature for 1 h to eliminate the background of binding to beads in the total protein. RNA was incubated with cell lysate for 1 hour and then incubated with beads coated with streptavidin. After washing the beads, add 30 μL 1 × protein sample buffer to the washed beads, mix them repeatedly, and heat them in boiling water for 10 min. After the sample passed western blot at 10% SDS-PAGE electrophoresis, the PTBP1 target band was detected.

### 2.7 MTT assay

6-10B and SUNE-1 cells were plated in 96-well plates and we used MTT assay to detect the cell viability. MTT (0.5 mg/mL; Beyotime Biotechnology, China) was added after curcumin treatment and incubated for 3 h at 37°C. And 150 μL DMSO was added and incubated for 15 min. We measured the absorbance at 490 nM.

### 2.8 Fluorescence *in situ* hybridization (FISH) assay

The probes of linc01513 were synthesized by RiboBio (Guangzhou, China), and FISH *in situ* hybridization kit (RiboBio, Guangzhou, China) was used for fluorescence staining. Nucleus was stained with DAPI. The pictures were observed and taken pictures with Olympus fluorescence microscope.

### 2.9 Transwell assay

24-well transwell (Corning, USA) with or without matrigel were used to investigate cell migration and invasion. 2 × 10^5^ 6-10B or SUNE-1 was seeded on the cell culture insert precoated with 1 µg/µL Matrigel (BD Biosciences, USA). Medium include FBS was used to stimulate invasion in the bottom of wells. After 48 h, the invasion cells were stained with a 0.1% crystal violet solution.

### 2.10 Wound-healing assay

6-10B and SUNE-1 were seeded in 6-well plates overnight, and then scratched using a 100 μL pipette tip. The scratch was visualized using phase-contrast microscopy (×4 objective) at 0, 24h.

### 2.11 lncRNA expression profiling and statistical analysis

LncRNA expression profiles of 16 patients with nasopharyngeal carcinoma were obtained from Gene Expression Omnibus (series accession: GSE61218). Welch's T-test was performed to test the means between adjacent normal and cancer samples. P values were adjusted by Benjamini-Hochberg (BH) procedure. LncRNAs with adjusted P values below 0.05 and absolute fold change values greater than 1.5 were considered as significantly differential expression. The statistical analyses were performed in using R Statistical Software.

### 2.12 qRT-PCR

Total RNA was extracted from cells or tissues using TRIzol reagent (Invitrogen, USA). RNA reverse transcription was performed using a reverse transcription kit (Thermo Fisher Scientific, USA). qRT-PCR was performed by QuantiTect SYBR Green PCR Kit (Qiagen, USA) to determine the relative levels of the linc01513 and PTBP1.

### 2.13 RNA immunoprecipitation (RIP)

RIP assays were performed using a Magna RIP kit (Millipore, USA) according to the manual. PTBP1 antibody (Protein-tech, China) was used for the RIP assay. IgG was used as a negative control.

### 2.14 Statistical analysis

Data were shown as mean±SD. Student's *t*-test or one-way ANOVA was used to compare the groups. P<0.05 was considered significance.

## 3. Results

### 3.1 Linc01513 was low expressed in nasopharyngeal carcinoma

**Figure 1:**
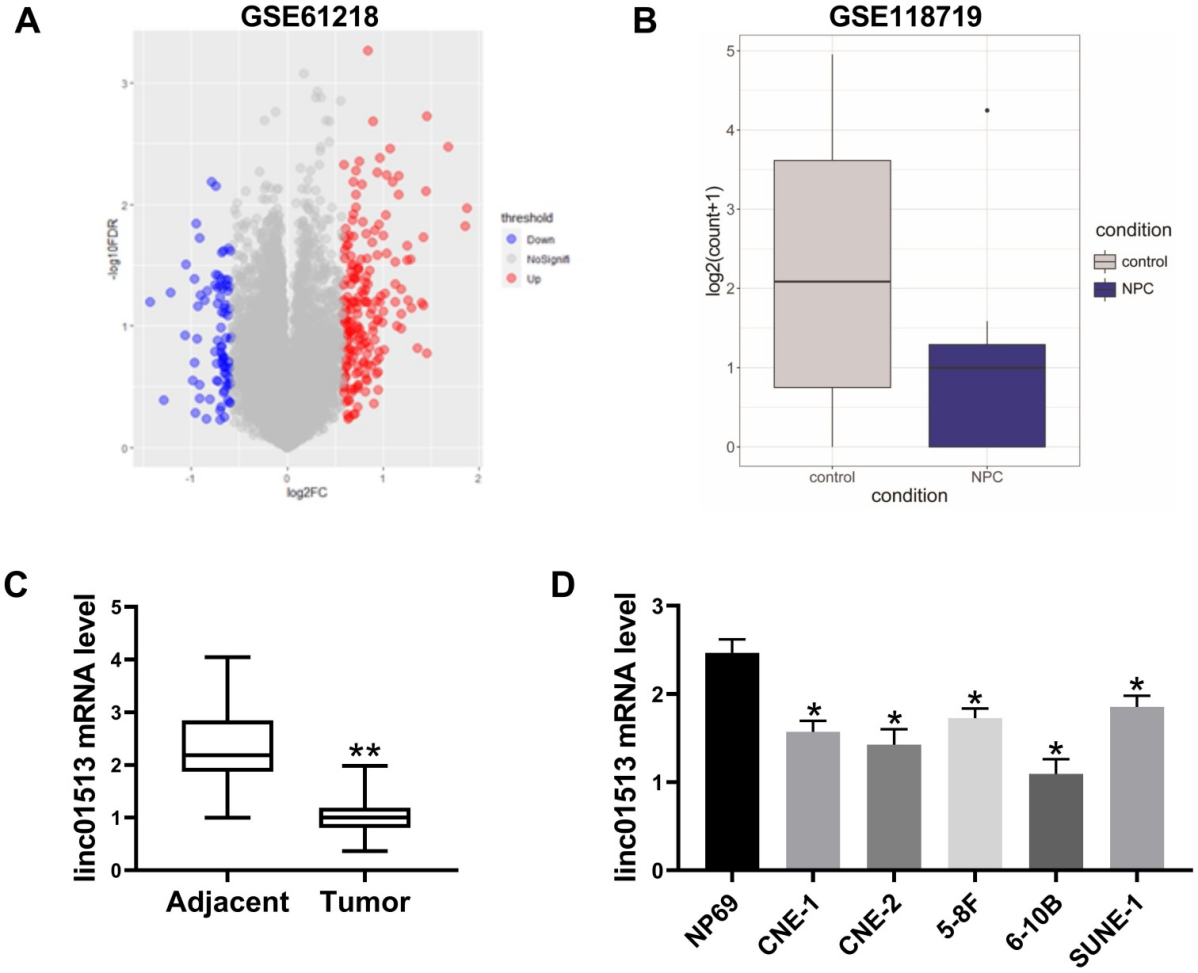
** Expression of linc01513 in nasopharyngeal carcinoma.** (A) LncRNAs expression profiles of 10 patients with nasopharyngeal carcinoma were obtained from Gene Expression Omnibus (series accession: GSE61218). (B) Linc01513 expression from Gene Expression Omnibus (series accession: GSE118719). (C) Expression of linc01513 in nasopharyngeal carcinoma and adjacent tissues determined by qRT-PCR. *n*=58; **p<0.01 *vs* adjacent tissues. (D) Expression of linc01513 in nasopharyngeal carcinoma cell lines and normal NP69 cell lines determined by qRT-PCR. *n*=3; *p<0.05 *vs* NP69.

In order to find lncRNA that plays a key role in nasopharyngeal carcinoma, we analyzed the GSE61218 data set, including 16 cases of nasopharyngeal carcinoma and 6 normal samples. T-test was performed for BH correction, and the significance threshold was set as FDR<0.05, |FC|>1.5 was the difference of lncRNAs. There were 84 down-regulated lncRNAs and 192 up-regulated lncRNAs, among which ENST00000416930 (linc01513) was significantly down-regulated in nasopharyngeal carcinoma (mean value: 1.361567 vs 3.6801102, FDR=0.00054) (Figure 1A). Furthermore, we analyzed the GSE118719 dataset and found that linc01513 was significantly down-regulated (Figure 1B).

In order to verify the expression of linc01513 in nasopharyngeal carcinoma, RNA was extracted from the nasopharyngeal tissue samples of 58 patients with definite pathological diagnosis and 58 adjacent nasopharyngeal epithelial tissue samples. QRT-PCR showed that the expression level of linc01513 in nasopharyngeal carcinoma tissues was significantly lower than that in normal nasopharyngeal epithelial tissue (Figure 1C). Furthermore, we determined linc01513 level in nasopharyngeal carcinoma cells CNE-1 and CNE-2, 5-8F, 6-10B, SUNE-1 and normal nasopharyngeal epithelial cells NP69. According to the results, linc01513 was low expressed in nasopharyngeal carcinoma cell lines, consistent with the tissue results (Figure 1D). And, the results indicated low relative linc01513 expression in 6-10B and high relative linc01513 expression in SUNE-1. Therefore, we finally selected 6-10B and SUNE-1 cells for follow-up study (Figure 1D).

### 3.2 Linc01513 inhibited the proliferation, invasion and migration of NPC cells

**Figure 2:**
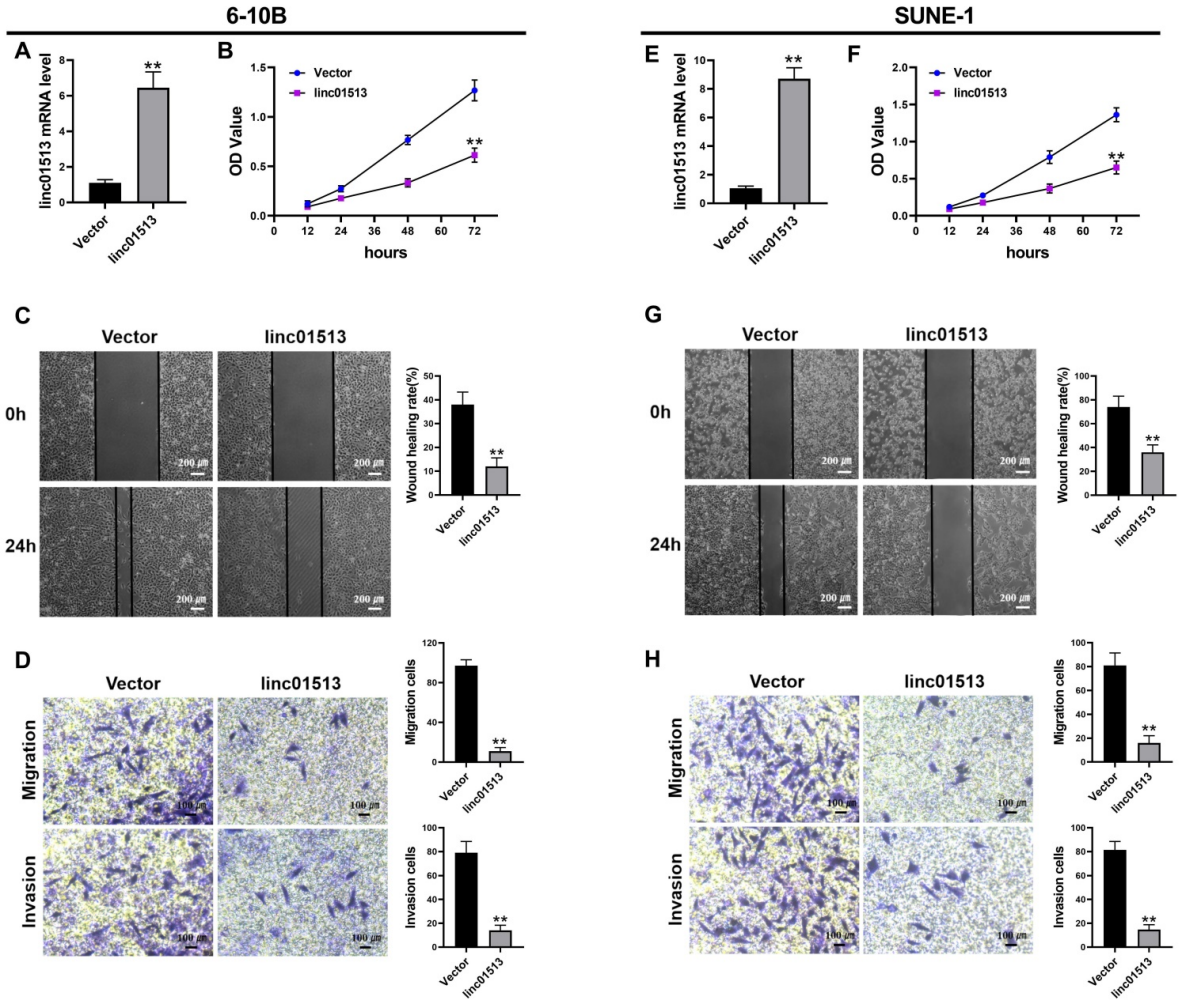
**Linc01513 regulated the proliferation, invasion and migration of NPC cells.** (A, E) Validation of linc01513 overexpression efficiency of stable transfected 6-10B and SUNE-1 cells by qRT-PCR. *n*=5; **p<0.01 *vs* vector group. (B, F) MTT was used to detect the effect of linc01513 overexpression on 6-10B and SUNE-1 cell proliferation ability. *n*=10; **p<0.01 *vs* vector group. (C, G) 6-10B and SUNE-1 migration ability was measured by wound healing assay after forced expression linc01513. Bar=200 uM; *n*=5; **p<0.01 *vs* vector group. (D, H) Transwell assay with or without matrix was used to detect cell migration or invasion of 6-10B and SUNE-1 after forced expression linc01513. Bar=100 uM; *n*=5; **p<0.01 *vs* vector group.

In order to test the effect of linc01513 on nasopharyngeal carcinoma cells, we constructed linc01513 stable overexpression cell lines and verified the transfection efficiency by qRT-PCR (Figure 2A, E). The effects of linc01513 on the proliferation, migration and invasion of 6-10B and SUNE-1 cells were verified by MTT experiment (Figure 2B, F), wound healing assay (Figure 2C, G) and transwell experiment (Figure 2D, H). The results showed that the proliferation, invasion and migration of 6-10B and SUNE-1 cells were significantly inhibited when linc01513 was forced expressed compared with the vector group.

### 3.3 Linc01513 inhibited the proliferation of NPC cells in xenograft model

**Figure 3:**
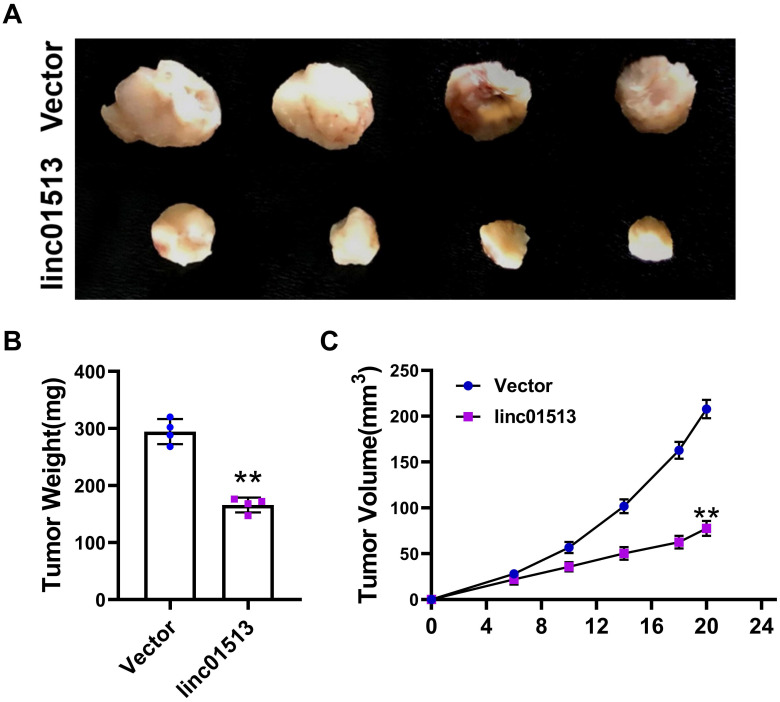
** Linc01513 inhibited tumor growth.** (A) Subcutaneous xenograft experiment results. *n*=4. (B) The tumor weight was measured for each xenograft. *n*=4; **p<0.01 *vs* vector group. (C) The tumor volume was measured using calipers in 0, 6, 10, 14, 18, 20 days. *n*=4; **p<0.01 *vs* vector group.

In order to further investigate the role of linc01513 *in vivo*, we used 6-10B cell lines with stable overexpression of linc01513 to establish a subcutaneous tumor-bearing model in nude mice. After 21 days, the size of the xenograft tumor was measured (Figure 3A). Tumor volume and weight were significantly reduced in mice overexpressed with linc01513 compared to the control group (Figure 3B, C). These results suggested that linc01513 inhibited the growth of nasopharyngeal carcinoma *in vivo*.

### 3.4 Linc01513 functions in combination with PTBP1

**Figure 4:**
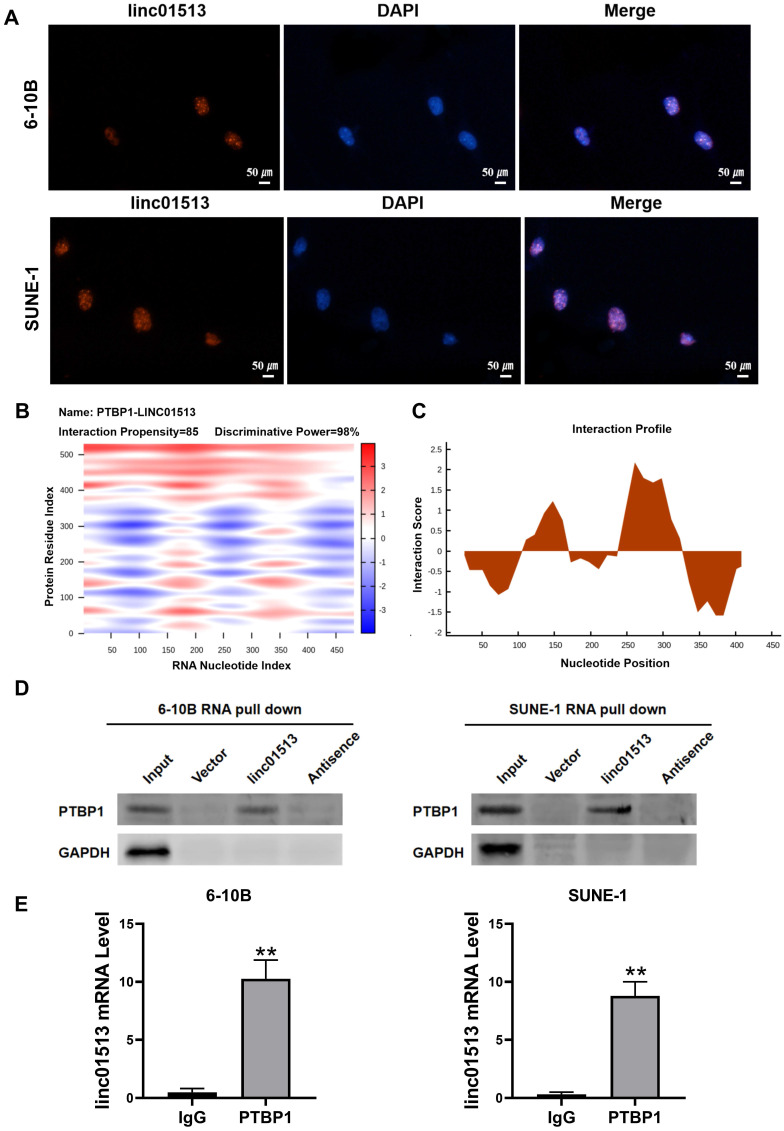
** Linc01513 binded to PTBP1.** (A) Localization of linc01513 was detected by FISH. Bar=50 uM. (B) RNA protein interaction Prediction of linc01513 with PTBP1 using the catRAPID graphic. (C) The interaction profile between PTBP1 protein and linc01513 predicted by catRAPID fragment. (D) Western blot analysis of RNA pull-down assay in 6-10B and SUNE-1 cell. (E) RIP-PCR analysis of interaction between PTBP1 and linc01513. *n*=3; **p<0.01 *vs* IgG group.

In order to explore the molecular mechanism of the antitumor effect of linc01513, we firstly analyzed its localization. FISH results showed that linc01513 was mainly localized in the nucleus (Figure 4A), suggesting that it may combine with nuclear proteins or molecules. Considering that many studies have confirmed that lncRNAs could combine with proteins to perform biological functions, we predicted linc01513 binding proteins by catRAPID, a web server for prediction of protein-RNA interactions 17. The database prediction results show that linc01513 may bind to PTBP1, a RNA binding protein (Figure 4B, C). RNA pull-down experiment was conducted to further verify that linc01513 could bind to PTBP1 protein (Figure 4D). In addition, the RIP-qPCR was used to confirmed the binding relationship between PTBP1 protein and linc01513 in 6-10B and SUNE-1 cells (Figure 4E). These results indicate that linc01513 binds to PTBP1 and may regulates the expression of PTBP1 to effect the tumour progression.

### 3.5 Linc01513 inhibited PTBP1 protein to alleviate EMT of NPC cells

**Figure 5:**
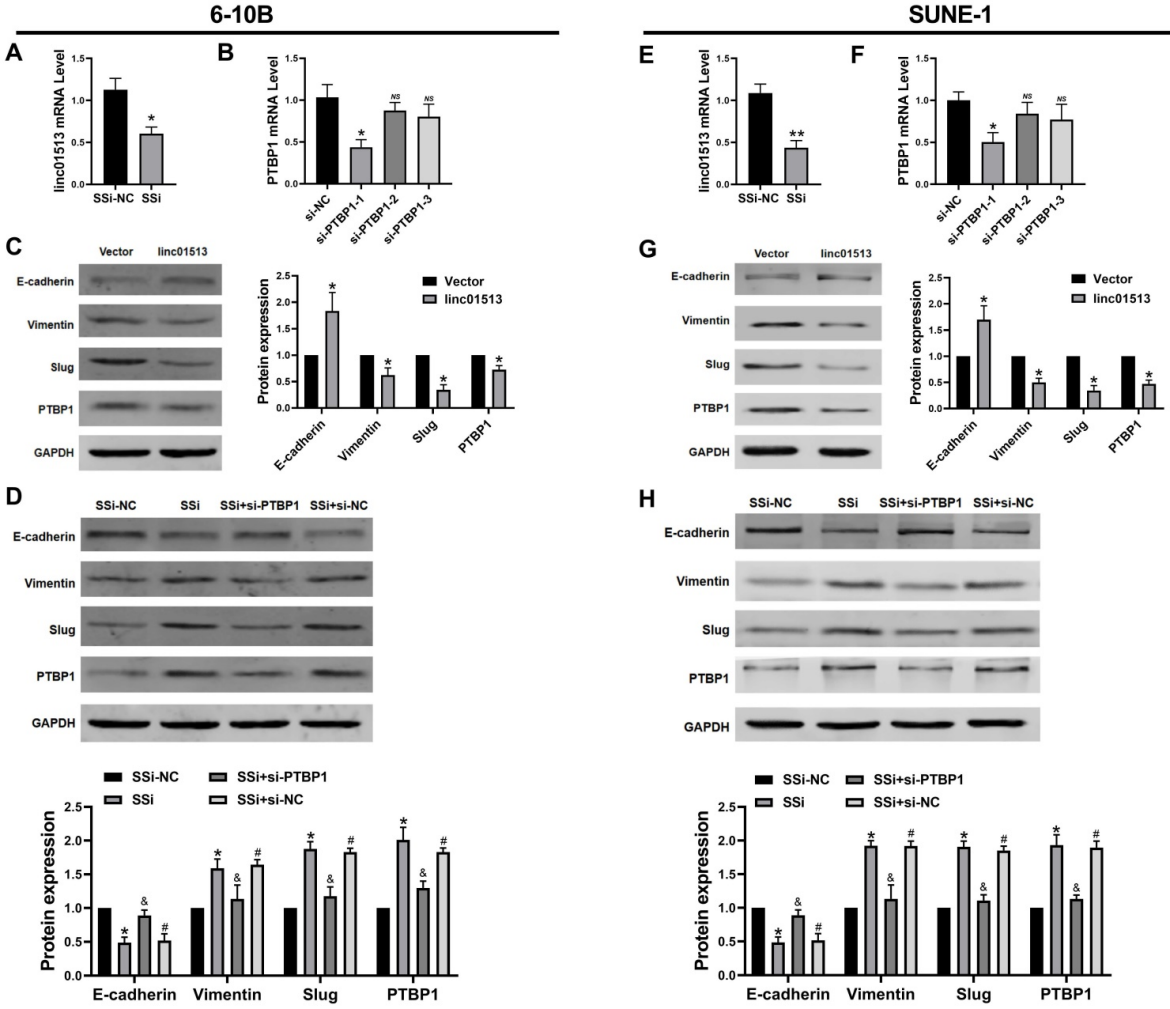
** Linc01513 regulated PTBP1 protein to affect the EMT.** (A, E) Smart Silencer efficiency detected by qRT-PCR to silence linc01513. *n*=5; *p<0.05 *vs* vector group. (B, F) Small interfering RNA (siRNA) of PTBP1 was constructed and screened using qRT-PCR. *n*=5; *p<0.05 *vs* si-NC; *ns,* no significant. (C, G) Western blot was used to measure the PTBP1 and EMT relative Slug, E-cadherin and vimentin protein level after overexpression linc01513. *n*=3; *p<0.05 *vs* vector. (D, H) Western blot was used to detect the PTBP1, Slug, E-cadherin and vimentin protein level transfected with linc01513 smart silencer and PTBP1 siRNA. *n*=3; *p<0.05 *vs* SSi-NC; &*p*<0.05 *vs* SSi; #*p*<0.05 *vs* SSi+si-PTBP1; SSi, smart silencer; si-PTBP1, Small interfering RNA of PTBP1.

Current studies generally believe that PTBP1 plays a pro-cancer role in a variety of tumors 18, 19. Firstly, we constructed the smart silencer of linc01513 and the Small interfering RNA (siRNA) of PTBP1 (Figure 5A, B, E, F). To explore whether the linc01513 regulates PTBP1 protein level to affect the EMT of NPC cells, we performed western blot to evaluate the PTBP1 and the maker of EMT. As shown in Figure 5C and Figure 5G, the expression of PTBP1, Slug and vimentin in NPC cells decreased after the up-regulation of linc01513 expression, while the expression of E-cadherin was increased. On the contrary, when the expression of linc01513 was down-regulated, the expression of PTBP1, Slug and vimentin in NPC cells was increased, while the expression of E-cadherin decreased. At the same time, after silencing the PTBP1, the increased changes in Slug and vimentin levels caused by down-regulating the expression of linc01513 were inhibited (Figure 5D, H). These results suggest that linc01513 may inhibit Slug expression and EMT progression in NPC cells through binding of PTBP1.

### 3.6 Knocking down PTBP1 partially reverse the tumor-promoting effect caused by downregulation of linc01513

**Figure 6:**
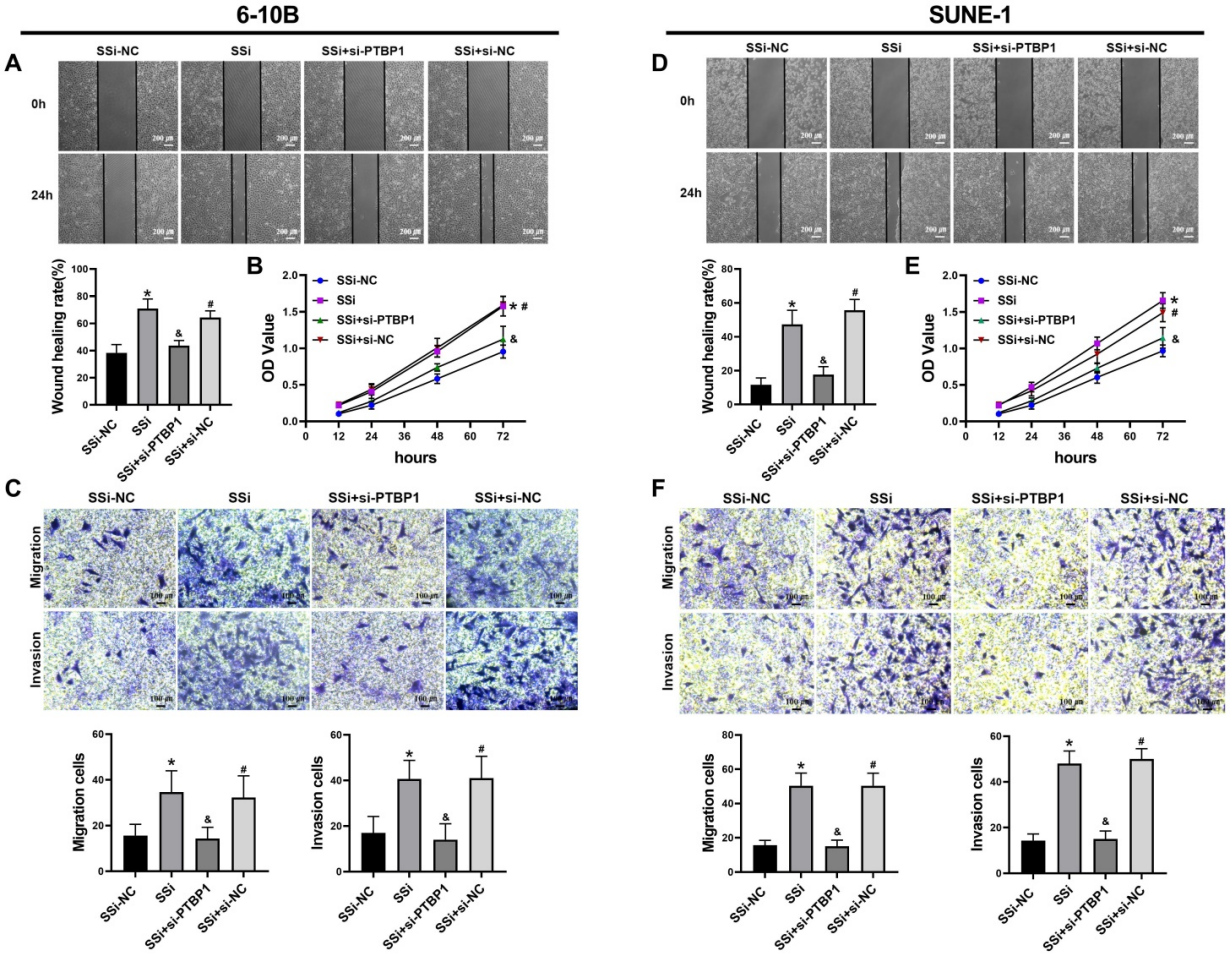
** PTBP1 was involved in the effects of linc01513 on the proliferation, migration and invasion of NPC cells.** (A, D) 6-10B and SUNE-1 migration ability was measured by wound healing assay after transfected with linc01513 smart silencer and PTBP1 siRNA. Bar=200 uM; *n*=5; *p<0.05 *vs* SSi-NC; &*p*<0.05 *vs* SSi; #*p*<0.05 *vs* SSi+si-PTBP1. (B, E) MTT was used to detect the effect of linc01513 smart silencer and PTBP1 siRNA on 6-10B and SUNE-1 cell proliferation ability. *n*=10; *p<0.05 *vs* SSi-NC; &*p*<0.05 *vs* SSi; #*p*<0.05 *vs* SSi+si-PTBP1. (C, F) Transwell assay with or without matrix was used to detect cell migration or invasion of 6-10B and SUNE-1 after transfected with linc01513 smart silencer and PTBP1 siRNA. Bar=100 uM; *n*=5; *p<0.05 *vs* SSi-NC; &*p*<0.05 *vs* SSi; #*p*<0.05 *vs* SSi+si-PTBP1.

We performed rescue experiments to further verify whether the regulatory effect of linc01513 on nasopharyngeal carcinoma cells is dependent on PTBP1. We simultaneously knocked down the expression of linc01513 and PTBP1 in 6-10B and SUNE-1 cells. Wound healing assay (Figure 6A, D), MTT (Figure 6B, E) and Transwell (Figure 6C, F) were used to verify the changes in migration, proliferation, and invasion ability of NPC cells. The results showed that the increased proliferation, invasion and migration of NPC cells induced by linc01513 smart silencer were partially reversed by knockdown of PTBP1. These results suggest that linc01513 can regulate the progression of nasopharyngeal carcinoma by inhibiting PTBP1.

## 4. Discussion

In this study, we found that the expression level of linc01513 in nasopharyngeal carcinoma tissues and cells was significantly decreased. Further functional study showed that linc01513 inhibited the proliferation, invasion and migration of nasopharyngeal carcinoma cells. In the mechanism, linc01513 may bind and inhibit PTBP1 protein to regulate the malignant progression of nasopharyngeal carcinoma.

LncRNA is an important regulatory factor in the progression of nasopharyngeal carcinoma 20, 21. LncRNA AFAP1-AS1 can regulate the integrity of cellular myofibroids and regulate cytoskeletal remodeling by changing the expression level of AFAP1 protein, thus promoting the invasion and metastasis of nasopharyngeal carcinoma cells 22. In addition, lncRNA FOXD1-AS1, RP11-624L4.1 and linc00669 play diverse roles in the occurrence and development of nasopharyngeal cancer 23-25. In this study, the GEO database dataset GSE61218 was used to screen and the linc01513 expression was significantly lower. Further experimental results showed that overexpression linc01513 inhibited the proliferation, metastasis and EMT progression of NPC cells *in vivo* and *in vitro*, suggesting that linc01513 played a role as a tumor suppressor in NPC. Therefore, it is very necessary to explore the molecular mechanism of linc01513 in inhibiting nasopharyngeal carcinoma.

It is well known that lncRNA can regulate the occurrence and development of tumors at various levels, such as chromatin modification, transcriptional regulation and post-transcriptional regulation, while the function of lncRNA is closely related to its subcellular localization 26. LncRNA CASC9 is mainly localized in the cell nucleus and can promote the expression of LAMC2 at the transcription level by directly binding to transcriptional coactivators 27. In order to obtain the subcellular localization of linc01513, we conducted FISH experiments, and the results showed that linc01513 was mainly localized in the nucleus in nasopharyngeal carcinoma cells, which suggested that linc01513 might bind to some nuclear proteins or molecules and play a role at the transcription level. We used catRAPID, a server for large-scale calculations of protein-RNA interactions, to predict the proteins that bind to linc01513. We focused on PTBP1, which has been shown to be associated with invasion, metastasis and poor prognosis of cancer. Then, we carried out RNA-pull down experiment and RIP-PCR to further verify the interaction of linc01513 and PTBP1.

Functionally, si-PTBP1 partially reverses the function of linc01513 smart silencer on the proliferation, invasion and migration of nasopharyngeal carcinoma cells. In addition, we found that linc01513 can inhibit the EMT process through PTBP1. In conclusion, the biological function and potential mechanism of linc01513 in the occurrence and development of NPC were preliminarily discussed, providing a theoretical basis for targeted therapy of NPC.

## 5. Conclusion

*5.1* Low expression of linc01513 in NPC tissues and cells.

*5.2 In vitro* and *in vivo* experiments showed that linc01513 inhibited the proliferation, migration and invasion of nasopharyngeal carcinoma cells.

*5.3* Linc01513 can mediate the EMT process and malignant biological behavior of NPC cells by binding with PTBP1, which provides a new molecular marker for the prognosis and treatment of NPC.

## Author Contributions

Juan Wang and Xiaolan Cai made substantial contributions to the conception and design of the study; Liqiang Zhang contributed to the acquisition, analysis and interpretation of the data; all authors participated in drafting the manuscript; Dapeng Lei revised it critically; all authors read and approved the final version of the manuscript.

## Funding

This research was funded by Key Research and Development Project of Shandong Province (2019GSF108265).

## Figures and Tables

**Table 1:** Clinicopathologic features of patients (n=58)

Characteristics	Value
Mean age (range), years Sex (male/female), n Differentiation (undifferentiated/differentiated), n Histology (squamous/others), n Lymph node metastasis (+/-), n Distal metastasis (+/-), n Clinical TNM stage (I-II/III-IV), n	46 (27-63) 39/19 31/27 58/0 32/26 0/58 36/22
